# Mutation-induced heme pocket destabilization in myoglobinopathy

**DOI:** 10.3389/fbinf.2026.1887793

**Published:** 2026-07-13

**Authors:** Sangita Kachhap, Ryan Sturms, Kiran Kumar Adepu, Vaidehi Ulaganathan, Patricia Jennings, Sree V. Chintapalli

**Affiliations:** 1 Duke Human Vaccine Institute, Duke University School of Medicine, Durham, NC, United States; 2 Department of Chemistry and Physics, Drake University, Des Moines, IA, United States; 3 Arkansas Children’s Nutrition Center, Little Rock, AR, United States; 4 Department of Pediatrics, University of Arkansas for Medical Sciences, Little Rock, AR, United States; 5 Department of Food Science Nutrition, Faculty of Applied Sciences, UCSI University, Kuala Lumpur, Malaysia; 6 Department of Chemistry and Biochemistry, University of California San Diego, San Diego, CA, United States

**Keywords:** heme, molecular dynamics simulation, mutation, myoglobin, myoglobinopathy

## Abstract

**Introduction:**

Myoglobin (Mb) is a heme-binding protein essential for oxygen storage, transport, and redox regulation, governed by a finely tuned network of electrostatic and hydrogen bonding interactions within the heme pocket. A point mutation His97Tyr leads to a hereditary disease, myoglobinopathy which disrupts heme-propionate interactions and leads to protein aggregation. Previous structural studies relied on a crystallographic model in which a Lys45Arg substitution was introduced to stabilize the heme pocket, masking the effects of the His97Tyr mutation and preventing clear interpretation of its role.

**Methods:**

To resolve this, we performed classical molecular dynamics simulations to systematically dissect the individual and combined structural effects of residues 45 and 97 by comparing the His97Tyr mutation in the native Lys45 (wild-type) and the crystallographic Arg45 residue.

**Results:**

Our results show that residue 45 plays a dominant role in regulating long-range dynamical networks, with Arg45 producing more spatially coherent correlations than the diffuse dynamics observed in the wild-type Lys45 protein. Structural variability within the heme pocket is primarily driven by redistribution of heme-propionates, where residue 45 acts as the main determinant, while residue 97 contributes an additive effect. Although iron coordination remains preserved across all systems, the wild-type His97Tyr variant exhibits tilting of the heme group within the pocket, indicating increased conformational flexibility.

**Discussion:**

Together, these findings suggest that the disease-associated mutation alters heme dynamics and highlights the critical role of sequence context in interpreting mutation-driven changes in myoglobin structure and function.

## 1 Introduction

Myoglobin (Mb) is a small, monomeric heme protein found in mammalian muscle tissue that adopts a globin fold formed by eight α-helices (A-H) and functions to reversibly bind O_2_ in a hydrophobic ligand-binding pocket (Kendrew et al., 1958). The heme prosthetic group is coordinated to the proximal histidine (His93) on the F helix by a coordinate covalent bond from the epsilon nitrogen. On the opposite face, the distal histidine (His64), on the E helix, occupies space near the sixth coordination site, thereby controlling ligand access and stabilizing bound O_2_ through hydrogen bonding (h-bond). The surrounding helical framework, especially the F-G region, helps position the heme and prevents solvent exposure through interactions between the heme propionate groups and residues of the helical framework. This well-structured architecture maintains optimal O_2_ storage, transport, and redox conditions (Kendrew et al., 1958; Ordway and Garry, 2004). Mb also facilitates O_2_ diffusion between the sarcolemma and mitochondria to maintain oxidative phosphorylation in response to varying metabolic demands (Wittenberg, 1970; Merx et al., 2001). In situations of enhanced workload or hypoxia, Mb releases O_2_ to fuel mitochondrial respiration and ATP generation (Taylor et al., 1986; Wittenberg and Wittenberg, 1987).

Mb is now recognized as a multifunctional protein involved in redox biology and cellular signaling (Wittenberg and Wittenberg, 2003;Gros et al., 2010;Adepu et al., 2024). Mb scavenges NO at high O_2_ tension in nitric oxide (NO) metabolism, acts as a nitrite reductase during hypoxia, and is involved in regulating bioavailability of NO implicated in mitochondrial respiration (Koivisto et al., 1997; Merx et al., 2005; Plotnikov et al., 2009; Quinting et al., 2021). Recent studies have broadened the functional range of Mb from only interactions with gaseous ligands to its interactions with metabolites, especially lipids (Sriram et al., 2008; Yamada et al., 2013; Shih et al., 2014; Chintapalli et al., 2015; Shih et al., 2015; Jue et al., 2016; Chintapalli et al., 2018; Armbruster et al., 2022). Biochemical and computational research have shown that oxy-Mb binds long-chain fatty acids and acylcarnitines in a chain length dependent fashion (Chintapalli et al., 2016; Jue et al., 2017). These interactions occur in the hydrophobic pocket beneath the heme prosthetic group, where Lys45 and Lys63 make electrostatic contacts with lipid headgroups while surrounding hydrophobic residues stabilize the lipid tail. Emerging evidence also suggests that Mb can interact with central metabolic intermediates like pyruvate and lactate (Giardina et al., 1996; Adepu et al., 2022a;b). This extended functionality is further supported by Mb expression in non-muscle tissues, such as brown adipose tissue, where it is associated with the regulation of lipid metabolism, mitochondrial activity, and thermogenic homeostasis (Blackburn et al., 2021; Christen et al., 2022).

Although the role of Mb-derived O_2_ in supporting cellular energy metabolism is well established, its precise contribution under varying physiological conditions remains incomplete. The compensatory mechanisms in Mb-deficient systems have revealed that Mb may play a role as a constituent of a wider system of O_2_-carrying processes (Garry et al., 1998; Garry et al., 2000; Park et al., 2019). However, inhibition or genetic elimination of Mb causes low O_2_ uptake, low work output, and detrimental changes to metabolic performance under stressful conditions (Godecke et al., 1999; Grange et al., 2001; Meeson et al., 2001; Schlieper et al., 2004). These observations suggest that Mb has a context-specific role and is especially significant during elevated metabolic demands or low O_2_ supply (Ono-Moore et al., 2021; Zoladz et al., 2024).

While previous *in vitro* and *in silico* mutational studies predicted that structural alterations may have severe pathological effects, native mutations that cause life threatening conditions are rarely observed (Tian et al., 1993; Scott and Gibson, 1997; Scott et al., 2001; Cohen et al., 2006; Cohen et al., 2008). However, recently identified myoglobinopathy, a rare inherited muscle disease caused by a missense mutation, His98Tyr (His97Tyr in PDB structure numbering) has been identified (Olive et al., 2019). This H97Y mutation changes heme-propionate interactions, destabilizes the local heme environment, and exposes Mb to solvent exposure, thereby promoting oxidative damage, impairing O_2_ binding, and facilitating protein aggregation. Structural and biochemical studies have shown that His97 is integral to the electrostatic and h-bond network around the heme, and mutation to tyrosine results in steric and electronic alterations that undermine the stability and redox properties of the heme (Peterson et al., 1998; Liong et al., 2001). The crystal structure (**PDB ID: 3RGK**) with a Lys45Arg substitution has served as a reference in structural and computational studies of myoglobinopathy in the past (Hubbard et al., 1990) to obtain a stable heme pocket. Since Lys45 interacts with lipid head groups and contributes to electrostatic interactions around the heme pocket, its substitution with Arg can alter the charge distribution, side-chain motion, and the relationship between the side chain at position 45 and the heme propionates. As a result, the specific effect of the His97Tyr mutation cannot be clearly evaluated.

Thus, in the present study, we considered Lys45 (WT) to evaluate the effect of the His97Tyr mutation and compared it with the Lys45Arg system. We performed classical molecular dynamics simulations and comprehensive trajectory analysis to clarify the effect of residue 97 on heme geometry, electrostatics, and ligand interactions. Our structural analysis shows that His97Tyr substitution redistributes heme propionate interactions, particularly those involving residues 45 and 97, leading to changes in heme-pocket conformational variability in an amino acid sequence-dependent manner. These results suggest a structural framework connecting disease-related disruptions of h-bond and electrostatic interaction networks and changes in heme dynamics. This work highlights the importance of sequence context in interpreting mutation-specific effects and advances our understanding of how structural dynamics, metabolite interactions, and redox stability are coupled in myoglobin.

## 2 Methods

### 2.1 Preparation of human oxy-Mb models and variant structure

Crystal structure of human Mb with a Lys45 to Arg mutation (**PDB ID: 3RGK**) (Hubbard et al., 1990) served as the initial structural template to maintain consistency with prior computational studies of myoglobinopathy (Olive et al., 2019). Using this template, we investigated the His97Tyr mutation, thereby enabling direct comparison with previously reported structural and dynamic observations. However, the Lys45Arg substitution present in the crystal structure does not reflect the native sequence. Additionally, the Ala110 substitution present in the 3RGK template was intentionally retained. The Cys110Ala mutation in 3RGK was not included to reflect disease biology, but rather to make the protein more stable, homogeneous and more suitable for crystallization. Mb from native humans has only one residue of Cys110, whose reactive thiol group may oxidize or participate in intermolecular crosslinking, creating heterogeneous or aggregated protein populations during protein purification or structural studies. For this reason, Cys110Ala is often employed on recombinant human Mb when performing structural and biophysical studies. To obtain the WT structure (Lys45), the Mutator Plugin tool of VMD v1.9.3 (Humphrey et al., 1996) was used to revert Arg45 to the native Lys45. Further, the His97Tyr substitution was introduced to both backgrounds to yield a total of four systems: WT, WT/His97Tyr, Lys45Arg, and Lys45Arg/His97Tyr. Due to the unavailability of human oxy-myoglobin structure, the coordinates of the bound O_2_ molecule were transferred to the heme iron center from sperm whale oxymyoglobin (**PDB ID: 1MBO**) after structural alignment (Phillips, 1980), as previously performed in our MD simulations using the equine Mb structure (Chintapalli et al., 2015; Chintapalli et al., 2016; Chintapalli et al., 2018). During the process, the geometry of the Fe-O_2_ bond and the h-bond between the O_2_ and the N^ε^ of the distal His64 residue were maintained. Following mutation and placement of the O_2_ ligand, all structures were visually examined to verify appropriate side chain orientations and the absence of steric clashes.

### 2.2 Molecular dynamic simulations

All four systems of Mb (WT, WT/His97Tyr, Lys45Arg, and Lys45Arg/His97Tyr) were simulated with NAMD v2.13 (Phillips et al., 2005; Melo et al., 2018). System preparation, visualization, and trajectory analyses were performed by using custom Tcl scripts in VMD v1.9.3, and CPPTRAJ (Humphrey et al., 1996; Roe and Cheatham, 2013). Each simulation was performed under isothermal isobaric (NPT) conditions with CHARMM 36 force field (Huang et al., 2017), along with compatible heme and ion parameter sets. In the physiological state of oxy-Mb, heme was modeled with O_2_ bound in the ferrous state (Fe^2+^-O_2_). The parameters and partial atomic charges of the heme-O_2_ complex were retrieved from Daigle et al. study, that were optimized using standard Ab initio quantum mechanical calculations (Daigle et al., 2009). A bonded model of the heme iron coordination geometry was used in which the heme iron is coordinated to the proximal His93 and the bound dioxygen molecule throughout the simulations. The pre-defined bonded parameters of the heme model were kept fixed for the Fe-N(His93) and Fe-O_2_ interactions and no other restraints were used in the production runs. Solvation of each oxy-Mb complex was carried out in an explicit TIP3P water box with periodic boundary conditions, with a minimum buffer of 10 Å between the solute and the box in all directions. Respective chloride (Cl^−^) and sodium (Na^+^) ions were added to neutralize all four systems to obtain an ionic strength of 150 mM. Temperature (300 K) and pressure (1 atm) were controlled by means of Langevin dynamics with a damping coefficient equal to 1 ps^-1^. The pressure was maintained by a Nose-Hoover Langevin piston mechanism, whereas the particle mesh Ewald (PME) method was used to calculate long-range electrostatic interactions. Short-range nonbonded interactions were calculated with a cutoff distance of 12 Å, and a switching function was applied from 10 Å.

All bonds that contained hydrogen atoms were fixed with the SHAKE algorithm (Elber et al., 2011), and a 2 fs integration time step was used along with periodic boundary conditions applied in all three dimensions. Before production runs, energy minimization of each system was performed for 10,000 steps to eliminate steric contacts, initially by steepest descent minimization followed by conjugate gradient minimization. After energy minimization, a total of 200 ns of production MD simulations was performed as multiple consecutive runs of up to 2 ns each, and atomic coordinates were recorded at time steps of 1 ps, producing trajectories. All four simulations were performed with the same protocols to enable direct comparison of structural and dynamical features across all four systems. The heme tilt angle was used to quantify the orientation of the heme group in relation to the protein matrix. A least-squares plane was first fitted to the porphyrin ring using the heme macrocycle atoms (NA, NB, NC, ND and porphyrin carbon atoms C1-C20), and the normal vector to this plane was determined. A reference axis representing the orientation of the F-helix was defined using the Cα atoms of residues 89 and 93. The tilt angle of the heme was then determined as the angle between the heme plane normal vector and the F-helix reference vector in CPPTRAJ using the vectormath module (Roe and Cheatham, 2013). Changes in this angle reflect alterations in the orientation of the heme macrocycle within the heme pocket and provide a measure of mutation-dependent modulation of heme dynamics.

## 3 Results

### 3.1 Differential backbone stability reveals increased flexibility in K45 variants

**FIGURE 1:**
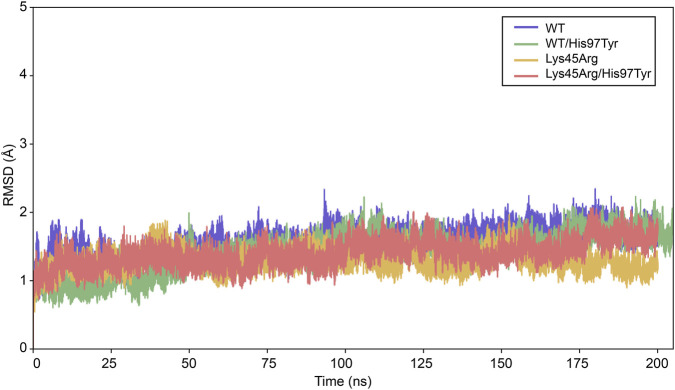
Backbone RMSD analysis of myoglobin variants. Time evolution RMSD of backbone atoms (N, Cα, C) for WT, WT/His97Tyr, Lys45Arg, and Lys45Arg/His97Tyr systems over the simulation trajectory.

Structural stability of the protein was estimated by following the root-mean-square (RMSD) deviation of the backbone with respect to the starting structure during the simulations (Figure 1). All the systems have a flattened RMSD at the end of trajectory, and RMSD values are distributed in a small range throughout the trajectory, which is in agreement with constant conformational sampling. The Lys45Arg system exhibits the lowest RMSD values fluctuating mainly in the range of ∼1.0–1.4 Å, which is indicative of a minimum amount of structural drift and stable backbone architecture. A similar situation is observed for the Lys45Arg/His97Tyr system, with RMSD fluctuations from ∼1.1 to 1.5 Å suggesting that the additional His97Tyr substitution does not substantially alter the global structural stability in the Lys45Arg background in accordance with the results published by Liong et al. (Liong et al., 2001). In contrast, the WT and WT/His97Tyr systems exhibit drift in frequency between ∼1.3 and 2.0 Å reflecting higher flexibility of the backbone in these systems. Although a slight increase is observed, the RMSD is not larger than about 2 Å in all systems, showing that the overall protein has not been destabilized structurally. These results indicate that although the Arg45Lys reversion brings with it a small but substantial change in backbone flexibility, the overall structure of all variants does not change significantly throughout the simulations. Based on this established structural stability, further analyses are focused on mutation-dependent internal dynamics and differences in communication at the residue level.

### 3.2 WT & WT/His97Tyr mutation interferes with long-range backbone correlated motions and communication networks

**FIGURE 2:**
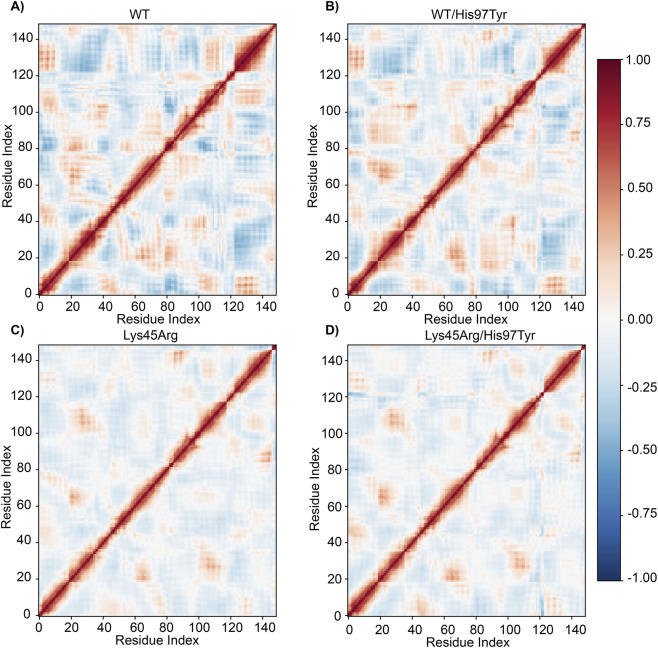
Backbone (N, Cα, C) atom distance cross correlation (DCCM). **(A)** WT, **(B)** WT/His97Tyr, **(C)** Lys45Arg, and **(D)** Lys45Arg/His97Tyr. Dynamic cross-correlation maps of backbone atoms fluctuations showing correlated (red) and anti-correlated (blue) motions between residue pairs. Differences in correlation patterns reflect mutation-dependent modulation of residue coupling and long-range dynamics. **A-helix:** Residues 3-18, **B-helix:** Residues 20-35, **C-helix:** Residues 36-42, **D-helix:** Residues 51-57, **E-helix:** Residues 58-76, **F-helix:** Residues 83-95, **G-helix:** Residues 100-118, **H-helix:** Residues 124-149.

To gain insight into the effect of substitutions at residues 45 and 97 on dynamics and long-range residue-residue communication in Mb, dynamic cross correlation matrix (DCCM) analysis was conducted using the backbone atoms (N, Cα, C and O) (Figure 2). This is an effective way to capture global dynamics and to compare long-range communication networks among all systems. The DCCM of the wild-type protein (Figure 2A) reveals a complex pattern of correlations, with regions of weak positive and negative correlations alternating throughout the matrix. These off-diagonal features reflect the presence of long-range coupling, but without extensive spatial contiguity, consistent with a diffuse and non-specific communication network. The pattern of the WT/His97Tyr system (Figure 2B) is similar, but with a slight redistribution of the correlations. The relative persistence of diffuse correlation features indicates that the substitution at residue97 has not perturbed the large-scale backbone correlation pattern. However, the Lys45Arg mutation causes significant redistribution of correlation strength in the DCCM. The correlation maps of the Lys45Arg single and Lys45Arg/His97Tyr double mutant (Figures 2C,D) systems are very similar. These maps are less diverse and have fewer separated pockets of anti-correlation when compared to the WT based systems, suggesting more uniform long-range dynamical propagation. The similarities between Lys45Arg and Lys45Arg/His97Tyr demonstrate that the mutation at residue 45 is the prominent factor in this change in dynamics, with the His97Tyr substitution introducing localized changes.

These effects are most pronounced in the Lys45Arg containing systems along helical segments of the B-C (residues 20-35 and 36-42), E-F (residues 58-76 and 83-95) and G-H (residues 100-118 and 124-149) regions. These helices are involved in more highly correlated motions in the Lys45Arg systems, suggesting strengthened long-range communication between secondary structure elements. In particular, the E-F helical segment, which includes the proximal heme pocket, displays different correlation patterns in the Lys45Arg systems compared to the WT system, implying enhanced or re-distributed dynamical correlations between the heme pocket and distant elements of the protein scaffold. These results suggest that residue 45 is an important contributor to backbone dynamics and long-range communication in Mb. These results suggest that rather than the WT protein providing a dynamically stable baseline, the replacement of Lys45 with Arg is associated with more coherent long-range correlations within the sampled trajectories. In contrast, changes at residue 97 have only limited influence on the global backbone correlated motions and do not appreciably alter the global communication network. These mutation-dependent differences in backbone dynamics may contribute to the distinct heme pocket conformational landscapes described in subsequent sections.

### 3.3 Mutation-specific landscapes of heme pocket conformational shifts are propionate driven

**FIGURE 3:**
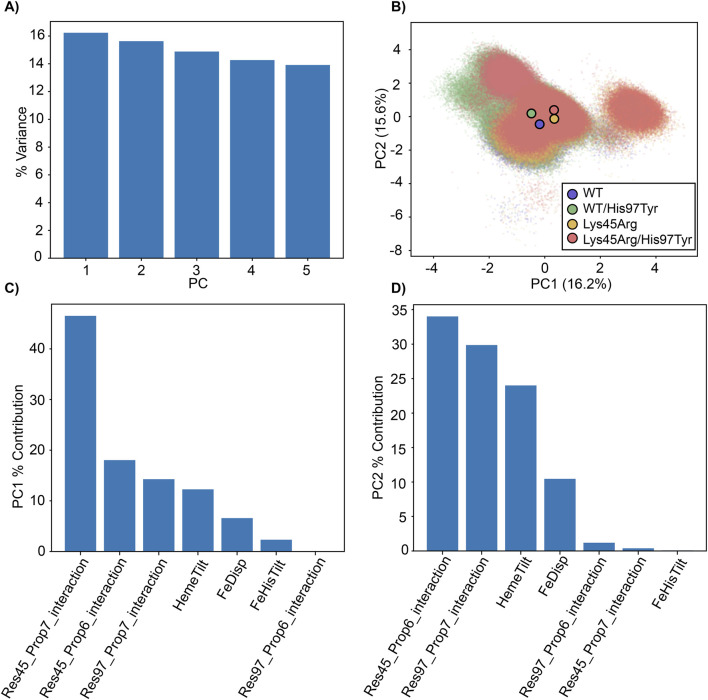
Principal component analysis for the heme pocket. **(A)** Percentage variance explained by the first five principal components (PCs). **(B)** Projection of simulation frames onto PC1-PC2 space, showing clustering of conformations for each system along with centroid positions. **(C)** Contribution of individual variables to PC1, highlighting dominant descriptors governing primary structural variation. **(D)** Contribution of individual variables to PC2, indicating secondary modes of variability.

To describe mutation-dependent variability in the heme pocket, principal component analysis (PCA) was carried out using descriptors of the heme environment, including residues 45 and 97 interaction with heme-propionate (with both propionates 6 and 7), as well as the heme tilt angle and iron (Fe) displacement. The first two principal components account for comparable but modest portions of the total variance (PC1: ∼16.2; PC2: ∼15.6) (Figure 3A), indicating that the conformational flexibility of the system is not one-dimensional, but has several collective motions. To evaluate whether these principal components reflect collective motions rather than diffusive sampling artifacts, cosine content analysis was performed for each trajectory. The cosine content values were low for both PC1 (0.0025-0.0882) and PC2 (0.0033-0.0371) across all systems (Table 1), indicating that the leading principal components are not dominated by random diffusion and instead capture coordinated structural variations within the heme pocket.

**TABLE 1:** Cosine content values for the first two principal components.

Systems	PC1	PC2
WT	0.0025	0.0086
WT/His97Tyr	0.0882	0.0371
Lys45Arg	0.0547	0.0055
Lys45Arg/His97Tyr	0.0116	0.0033

The projection of the trajectories on PC1-PC2 space exhibits clear clustering behavior in the four systems. The Lys45Arg and Lys45Arg/His97Tyr variants are localized within the same region (Figure 3B), indicating that the His97Tyr mutation in the Lys45Arg background does not substantially perturb the prevailing conformational landscape of the heme pocket. By comparison, in the Arg45Lys reversion systems, there is a definite PC1-shift (Figure 3B), demonstrating that perturbation at residue 45 fundamentally alters the dominant mode of structural variability. The WT/His97Tyr system shows a wider distribution than WT, indicating that the His97Tyr mutation enhances conformational heterogeneity without shifting the system toward the Lys45Arg conformational space. Analysis of variable contributions reveals that PC1 has dominant propionate interactions near residue 45 with Res45-Prop7 making the largest contribution and Res45-Prop6 at 46% (Figure 3C). The propionate interactions near residue 97 are minor contributors to PC1, with Res97-Prop7 interaction contributing moderately 14%, and Res97-Prop6 interaction contributing irrelevantly 0.007%. Conversely, PC2 reflects contributions from both propionate interactions and heme geometry with Res45-Prop6, Res97-Prop7 and Fe-heme tilt making the three largest contributions at 34, 29, and 24% respectively (Figure 3D), indicating a coupling between local interaction networks and global heme orientation. Fe displacement and Fe-His tilt have minimal contribution to both PC1 and PC2. These findings indicate that mutation-induced alterations in propionate interaction networks are the main determinants of heme-pocket conformational variability, with residue 45 occupying the dominant role (PC1), and residue 97 a secondary one (PC2).

### 3.4 Rewiring of heme propionate hydrogen bonds with mutation

To further characterize the heme pocket interactions identified by PCA, h-bond analysis was performed. Consistent with the PCA results, interactions between residues and propionates emerged as the strongest determinants of conformational variability in the heme pocket, revealing a network of h-bonds that were reorganized upon mutation (Table 2). In the Lys45Arg system, the propionate-6 (O1D and O2D) is anchored by the formation of persistent hydrogen bonds with Arg45 (occupancy >50%). In contrast, interactions between Arg 45 and propionate-7 (O1A and O2A) are weak (<4%), and contributions from Ser92 and His97 are negligible. A similar pattern is observed in the Lys45Arg/His97Tyr system, though Arg45-propionate-6 occupancy is somewhat reduced (41%-47%). The H97Y mutation introduces new Tyr97/propionate-7 interactions, but these occur with relatively low occupancy (∼5%), and Arg45 remains the primary stabilizing interaction. In the WT system, a decrease in propionate-6 h-bonding with residue 45 (28%-32%) is observed, as well as nearly complete elimination of contact with propionate-7 (<1%), resulting in a considerable loss of propionate anchoring. These changes in hydrogen bonding reflect the reduced h-bond donor capacity of Lys relative to Arg, as well as the differences in their side chain geometry. In the WT/His97Tyr system, a notable redistribution of h-bonds is observed. Although Lys45 only has moderate interactions with propionate-6 (∼35% occupancy), Tyr97 forms stronger hydrogen bonds with propionate-7 than those observed in the Lys45Arg/His97Tyr system, especially with O2A (∼10.9%) and O1A (∼3.4%). This suggests that the H97Y mutation partially compensates for the lost Lys45 interactions at propionate-6 through enhanced interactions at propionate-7, thus providing an alternative stabilization structure for the heme pocket. These results confirm the PCA findings and demonstrate that mutations in positions 45 and 97 do not merely weaken the interactions within the heme pocket but redistribute the contributions of h-bond interaction throughout the pocket. Specifically, the intricate role of residue 45 in regulating propionate-6 interactions (captured in PC1) and the secondary role of residue 97 in regulating propionate-7 interactions (captured in PC2) provide a mechanistic explanation for the conformational variability observed in the PCA.

**TABLE 2:** Hydrogen bond analysis of heme propionates with Mb.

Residue	Prop6_O1D	Prop6_O2D	Prop7_O1A	Prop7_O2A
% Lys45Arg - propionate h-bond				
ARG 45	51.43	58.07	3.49	3.88
SER 92	0.0	0.0	0.85	1.06
HIS 97	0.0	0.0	0.0	0.0
% Lys45Arg/His97Tyr - propionate h-bond				
ARG 45	46.61	41.1	3.82	3.79
SER 92	0.0	0.0	0.0	0.75
HIS 97	0.03	0.02	5.35	5.09
% WT - propionate h-bond				
LYS 45	32.09	28.24	0.32	0.1
SER 92	0.0	0.0	0.08	0.02
% WT/His97Tyr - propionate h-bond				
LYS 45	34.98	35.71	0.2	0.12
SER 92	0.0	0.0	0.5	0.23
TYR 97	0.03	0.02	3.42	10.91

### 3.5 Redistribution of propionate interactions regulates heme orientation and dynamics without altering protein core geometry

**FIGURE 4:**
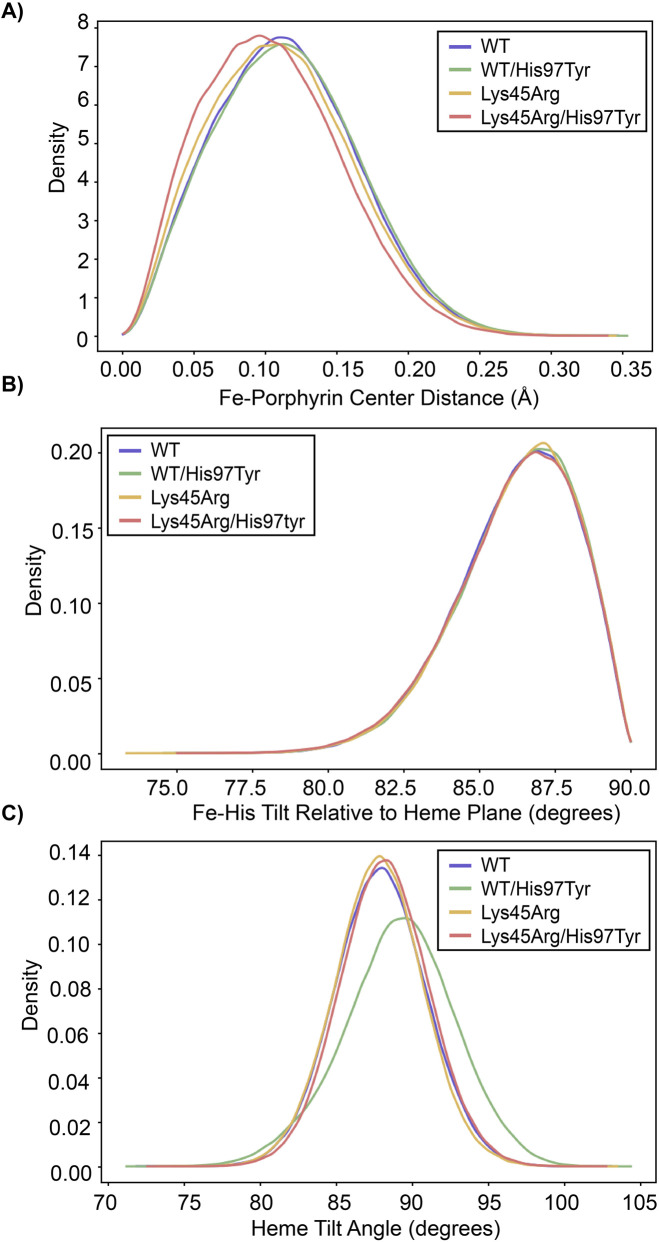
Distributions of Fe-porphyrin distance, Fe-His tilt, and heme tilt. **(A)** Distribution of Fe-porphyrin center distance. **(B)** Distribution of Fe-His tilt relative to the heme plane. **(C)** Distribution of heme tilt angle.

**FIGURE 5:**
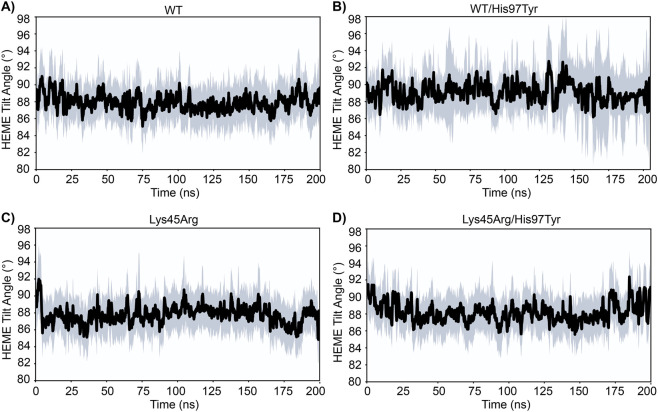
Time series moving average HEME tilt angle in Myoglobin variants. **(A)** WT, **(B)** WT/His97Tyr, **(C)** Lys45Arg, **(D)** Lys45Arg/His97Tyr. The black trace represents the moving average of the heme tilt angle, while the gray shaded region indicates instantaneous fluctuations sampled during the trajectory.

**FIGURE 6:**
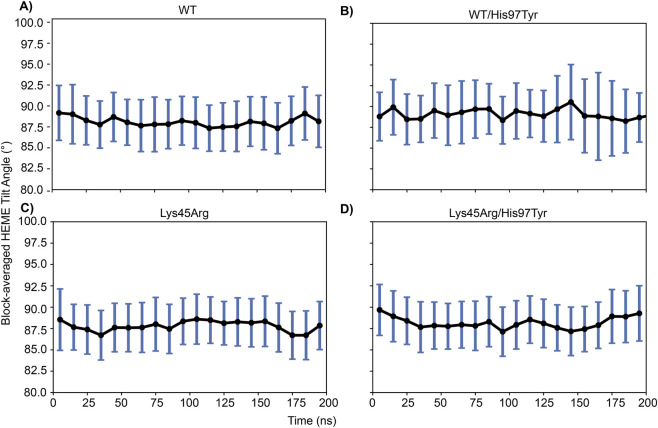
Block-averaged heme tilt angle in myoglobin variants. **(A)** WT, **(B)** WT/His97Tyr, **(C)** Lys45Arg, and **(D)** Lys45Arg/His97Tyr. The 200 ns trajectories were divided into consecutive 10 ns blocks, and the mean heme tilt angle was calculated for each block. Black circles represent the block-averaged heme tilt angle, and error bars represent the standard deviation of tilt angles sampled within each block. The block-averaged profiles reveal stable heme tilt dynamics throughout the simulations and demonstrate that the enhanced tilt fluctuations observed in the WT/His97Tyr variant are maintained across the entire trajectory rather than arising from transient events.

To further quantify the changes in the heme pocket dynamics due to mutation, we measured the displacement of Fe relative to the porphyrin plane, the Fe-His bond angle and the heme tilt angle (Figures 4, 5). These metrics collectively characterize heme geometry and indicate that the position of the Fe and Fe-His bond angle is tightly constrained across all systems. The Fe-porphyrin distance has a unimodal distribution, with the largest frequency found in the range of 0.09–0.12 Å (Figure 4A), indicating that Fe remains close to the porphyrin plane in all simulations. Notably, the Lys45Arg/His97Tyr system shows a narrower distribution of Fe-porphyrin distance (Figure 4A) than the other systems, which have a broader, overlapping distribution. Fe-His bond angle distributions are also preserved in all the systems and have narrow distributions around 87°-88° (Figure 4B). This overlap indicates that the axial coordination geometry of the heme Fe is conserved across all systems, consistent with being identified as a minor contributor in the dominant PCA modes. In contrast to the conserved Fe-centric parameters, mutation-dependent differences are pronounced in the heme tilt angle. The probability distributions of the tilt angle (Figure 4C) demonstrate that while each system displays a major population between ∼87° and 89° tilt, the WT/His97Tyr variant has a significantly wider distribution with a clear shoulder extending toward higher tilt angles (∼95°). This feature is absent in the WT, Lys45Arg, and Lys45Arg/His97Tyr systems, which display narrow, highly overlapping distributions indicative of more restricted heme orientations. Importantly, analysis of the time-resolved, moving average-smoothed plots of the heme tilt angle (Figure 5) demonstrates that the enhanced flexibility observed in the WT/His97Tyr system does not result from rare, transient excursions. Instead, an increase in tilt fluctuations is apparent early in the simulations and is sustained throughout the entire trajectory, with continued sampling of higher tilt angles (typically ∼86°–92°) and numerous excursions to even higher tilt angles (typically ∼94°–96°), compared with the other variants. This is consistent with His97Tyr induced disruptions of heme environment, including altered side-chain contacts and packing around the heme, resulting in enhanced flexibility and sampling of higher tilt angles. In contrast, the WT, Lys45Arg and Lys45Arg/His97Tyr variants display more constrained tilt dynamics as a function of time, with reduced fluctuations around a stable mean (∼87°–89°) and a narrower range of tilt angles sampled (∼85°–91°) (Figure 5). The combined distributional (Figure 4C) and time-resolved (Figure 5) analysis show that the His97Tyr mutation in the WT system results in a persistent enhancement of heme orientation dynamics. To better evaluate the stability and convergence of the heme tilt dynamics, block-averaging analysis was carried out by splitting the trajectories into overlapping time blocks and computing the average value of the tilt angle and the standard deviation in each (Figure 6). Block-averaged profiles did not show any systematic drift or systematic changes in the sampled tilt angles during the 200 ns simulations performed on all systems. Similar to the probability distributions and moving-average analysis, the average tilt angle for the WT/His97Tyr system was consistently greater than the other three systems, and the fluctuations in block-to-block tilt were larger in the WT/His97Tyr than in the other three systems. These observations suggest that the increased heme tilt dynamics for the His97Tyr substitution is not limited to a small fraction of the simulated trajectory, but instead has occurred consistently throughout the simulation, which increases confidence in the observed differences between the two mutations. These observations suggest that heme tilt may serve as a sensitive indicator of mutation-dependent modifications in propionate anchoring within the heme pocket.

### 3.6 Structural basis for altered heme orientation

**FIGURE 7:**
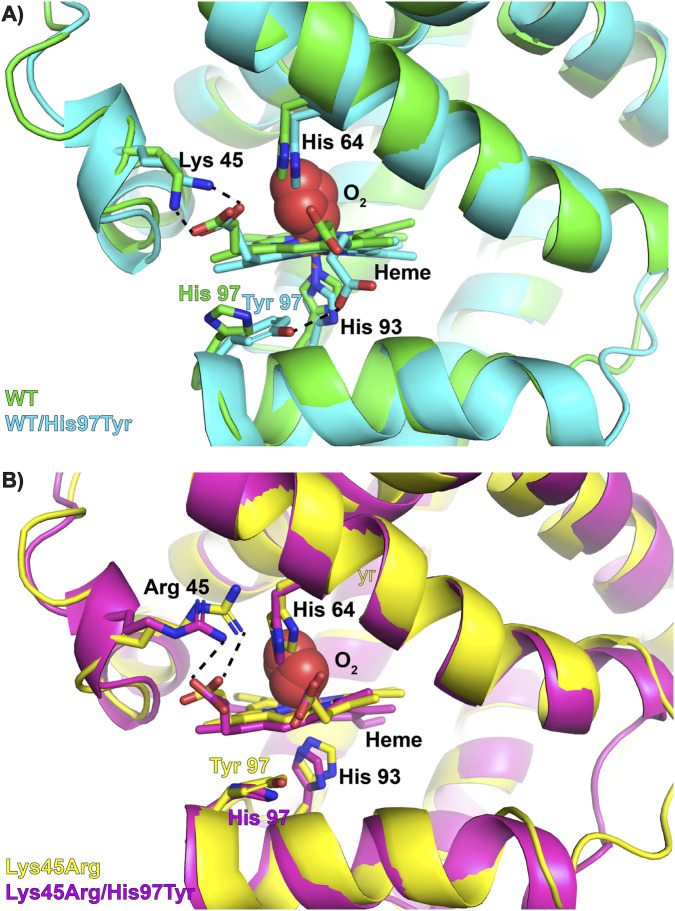
Structural basis for mutation-dependent modulation of heme-propionate interactions. Representative snapshots showing interactions among residue 45, 97, and heme propionate. **(A)** WT/Lys45 background, where His97Tyr disrupts local packing and reduces constraint on the heme propionates. **(B)** Lys45Arg background, where Arg45 forms stronger interactions with the heme propionates, helping maintain a more constrained heme environment even with His97Tyr. Dashed lines indicate h-bond or electrostatic interactions.

To gain additional insights into the structural basis for these mutation-specific changes in heme orientation, we compared representative structures of the heme pocket (Figure 7). In the WT/Lys45 system (Figure 7A), the heme propionate groups are constrained by relatively weak, transient electrostatic interactions, and mutation of His97Tyr alters the local h-bond and side chain packing network surrounding the heme. This results in fewer constraints on the propionate groups, allowing greater freedom of heme motion, consistent with the broader tilt distributions and enhanced fluctuations observed in Figures 4C, 5. However, in the Lys45Arg background (Figure 7B), the Arg45 residue forms more stable electrostatic and h-bond interactions with the heme propionates, anchoring in place even in the presence of the His97Tyr substitution. This constrains the mutation-induced effects on heme tilt, leading to the smaller distributions and fluctuations observed in these systems. These structural insights offer a potential molecular explanation for the observed dynamic differences in heme tilt and suggest that regulation of heme-propionate interactions at position 45 is a key determinant of mutation-induced changes in heme flexibility.

## 4 Discussion

In the present work, we analyzed the structural implications of the His97Tyr mutation of Mb in both experimentally relevant (Lys45Arg) and physiologically relevant (Lys45) backgrounds. Our principal finding is that the native Lys45 background exhibits markedly different structural behavior compared to the Arg45 (3RGK) mutant (Hubbard et al., 1990). Previous structural and biochemical analyses of sperm whale Mb have shown that mutation of His97 does not alter the overall protein structure but does result in slight heme perturbations which may increase susceptibility to heme dissociation (Olive et al., 2019). These observations led to the conclusion that His97 plays a major role in heme retention without causing significant structural rearrangements. Nonetheless, we find that this apparent structural resilience may be background dependent. In human Mb, the His97Tyr mutation in the native Lys45 background is associated with greater conformational flexibility in the heme environment, suggesting that the structural impact of the pathogenic mutation (His97Tyr) may not be fully captured by previous works using the Arg45 background (Olive et al., 2019).

Rather than simply resulting in heme distortion, the WT/His97Tyr system alters the entire interaction network governing heme stability. In sperm whale Mb (which has a native Arg45), His97 forms a stabilizing h-bond with the heme-propionate and contributes to restricting solvent access to the hydrophobic proximal pocket. Mutation of His97 in this sperm whale was shown to disrupt these interactions and open solvent access channels to the heme pocket facilitating heme loss without inducing substantial structural changes (Liong et al., 2001). However, our MD simulations of human Mb with a native Lys45 environment suggest more extensive consequences. The His97Tyr mutation in the native background not only disrupted electrostatic interactions in the heme pocket but also showed increased structural flexibility and altered long-range correlations within the heme environment. This change was accompanied by an increase in heme tilt, propionate mobility, and increased solvent exposure, effects that are suppressed in the human Lys45Arg background. The difference between Lys and Arg at position 45 is therefore critical to modeling dynamics in the human His97Tyr disease state. Whereas Arg45 imposes stricter electrostatic architecture, which partially constrains and compensates for the loss of His97 interactions, the native Lys45 allows a more dynamic environment in which the structural consequences of His97Tyr are more apparent. These findings extend the existing model of myoglobinopathy by suggesting a structural connection between mutation-induced perturbations in the native human sequence background and the experimentally observed structural conditions associated with increases oxidative susceptibility (Olive et al., 2019), narrowing the gap between molecular structure and disease phenotypes.

The structural perturbations identified in our simulations may have implications for lipid processing, potentially connecting with important pathological aspects in patients with myoglobinopathy (Olive et al., 2019; Hama et al., 2022; Hofbauer et al., 2022). For instance, residues Lys45 and Lys63 have been implicated in the coordination of long-chain fatty acids and acylcarnitines via electrostatic interactions with their polar headgroups (Chintapalli et al., 2015; Chintapalli et al., 2016; Chintapalli et al., 2018). Given the established role of these lysine residues, the change in the geometry of the heme and the enhanced dynamics of surrounding helices in the human WT/His97Tyr system suggest a possible structural remodeling of the lipid-binding. Specifically, heme propionate reorientation and local effects of structural perturbations could affect the positioning and stability of Lys-mediated lipid interactions, potentially influencing lipid binding efficiency or substrate specificity. This hypothesis, supported by the increased dynamics in our simulations, is consistent with the patient biopsy results, which revealed oxidation and deposition of lipids as sarcoplasmic bodies. Altered lipid interactions with mutant Mb may contribute to intracellular lipid dysregulation, which may favor oxidative lipid damage, the development of characteristic inclusions, and progressive fatty degeneration of skeletal muscle, which are all observed clinically in myoglobinopathy patients (Olive et al., 2019; Hama et al., 2022; Hofbauer et al., 2022).

Our findings provide a possible structural explanation for the dysregulation of gaseous ligand handling and metabolic strain observed in myoglobinopathy. In physiological conditions, Mb functions as a tightly controlled O_2_ buffer with ligand binding at the sixth coordination site of the iron affecting overall Mb stability (Kendrew et al., 1958). Experimental work has, however, indicated that during conditions mimicking hyperlactatemia O_2_ release and iron oxidation are enhanced (Adepu et al., 2022b). This lactate-induced effect may be further amplified by the structurally destabilized WT/His97Tyr mutation. The combination of an increased heme tilt, augmented solvent exposure, and decreased propionate interactions identified in these simulations may reduce structural constraints associated with ligand binding, potentially increasing susceptibility to altered O_2_ release kinetics even in physiological conditions. Such dysregulated O_2_ kinetics may compromise O_2_ delivery during activity, contributing to the energetic inefficiency and premature muscle fatigue characteristic of myoglobinopathy. Moreover, the mutant protein has been reported to exhibit increased heme lability and oxidative susceptibility, and the enhanced heme pocket dynamics observed here may provide a structural context for the increased oxidative susceptibility and protein aggregation reported experimentally in affected patients muscle tissue (Hofbauer et al., 2022).

Taken together, our computational results indicate the need to assess disease-related mutations in their native structural and electrostatic environment. Although the human Mb (Lys45Arg) background has been valuable in elucidating structural details, we have shown that this change causes stabilizing interactions that partially moderate the structural and dynamical effects of the His97Tyr mutation. Our simulations in the WT/His97Tyr environment identified mutation-induced structural changes in the heme pocket, including a change in heme tilt distribution and heme-propionate contacts with an increase in local protein dynamics. These structural characteristics are consistent with reduced heme stabilization. While the current simulations did not explicitly examine ligand binding and O_2_ release, the structural changes observed are consistent with a proposed mechanism of the mutation’s effect on heme stability, ligand binding, and O_2_ regulation. These observations suggest a potential connection between the molecular-level structural perturbations identified here and the clinical manifestations of myoglobinopathy. Additionally, because muscle lipid utilization is strongly influenced by dietary fatty acid availability and metabolic state, disruption of the Lys45-centered lipid-binding environment by the His97Tyr mutation may influence lipid handling and O_2_-dependent energy metabolism in a nutritionally sensitive manner. These findings also highlight the need for complementary investigations and experimental studies measuring heme loss, lipid interactions, and O_2_ kinetics during hyperlactatemia in the native human sequence.

Despite the sensitivity of the long-range correlated motions and the communication networks of residues, the main conclusions of this study are supported by several complementary analyses, such as heme tilt distributions, time-resolved heme dynamics, Fe-porphyrin displacement, Fe-His geometry and heme-propionate interaction networks. Importantly, the increased heme flexibility found in the native Lys45/His97Tyr system is maintained throughout the simulations and not caused by fluctuations that happen only on a smaller time scale. Moreover, the simulation protocol employed here has been successfully used in our previous studies of myoglobin-lipid interactions (Chintapalli et al., 2015; Chintapalli et al., 2016), lipid entry pathways (Chintapalli et al., 2018), and myoglobin-membrane interactions (Anishkin et al., 2023), where the observed conformational behavior was consistent with experimental findings. Despite the trends observed in the distributions of tilt angles at the heme, time-resolved heme dynamics, displacement of Fe from the porphyrin, geometry of Fe-His, and heme-propionate interaction networks from these mutation-associated analyses, we recognize that the present study is based exclusively on a single trajectory for each system. The additional convergence analyses presented here, including cosine-content measurements and block-averaged heme tilt profiles, assess sampling quality within individual trajectories but do not quantify between-trajectory variability. Future work with additional replica simulations using different velocity sets and longer trajectories, and with integration of data from continuing biophysical experiments on recombinant variants of human myoglobin, will be useful for further evaluating the statistical robustness of the comparative differences found here. The present simulations employed a fixed-charge classical force field representing the ferrous oxy-myoglobin state. Consequently, changes in iron oxidation state, electronic structure, ligand dissociation kinetics, heme release, and auto-oxidation were not explicitly modeled. Instead, the observed changes in heme tilt, solvent exposure, heme-propionate interactions, and conformational dynamics provide structural insights into how the His97Tyr mutation may influence these processes. Future QM/MM simulations and enhanced-sampling free-energy methods will be necessary for the direct simulation of redox chemistry, transitions between the different spin states of Fe, and O_2_ dissociation energetics.

## 5 Conclusion

This study demonstrates that the His97Tyr mutation observed in human myoglobinopathy has a much stronger structural and dynamic impact in the native Lys45 background than was apparent from prior studies using the Lys45Arg background of the 3RGK crystal structure. These findings underscore the importance of background context in determining the structural consequences of disease-associated mutations and highlight the necessity of physiologically relevant models for accurately interpreting pathogenic mechanisms.

## Data Availability

The datasets generated during this study are available in Zenodo at DOI: 10.5281/zenodo.20328402. README text file listed in the Zenodo archive explains the contents and structure of the folder. Separate folders are created for every simulated system with the associated PDB, PSF and compressed DCD files, and a specific folder contains the various topology, parameter, and simulation input files to reproduce the simulations.
